# Optimally-weighted non-linear beamformer for conventional focused beam ultrasound imaging systems

**DOI:** 10.1038/s41598-021-00741-5

**Published:** 2021-11-03

**Authors:** Anudeep Vayyeti, Arun K. Thittai

**Affiliations:** Biomedical Ultrasound Laboratory, Department of Applied Mechanics, Indian Institute of Technology, Madras, Chennai, India

**Keywords:** Engineering, Biomedical engineering, Electrical and electronic engineering

## Abstract

A novel non-linear beamforming method, namely, filtered delay optimally-weighted multiply and sum (F-D*ow*MAS) beamforming is reported for conventional focused beamforming (CFB) technique. The performance of F-D*ow*MAS was compared against delay and sum (DAS), filtered delay multiply and sum (F-DMAS), filtered delay weight multiply and sum (F-D*w*MAS) and filter delay Euclidian weighted multiply and sum (F-D*ew*MAS) methods. Notably, in the proposed method the optimal adaptive weights are computed for each imaging point to compensate for the effects due to spatial variations in beam pattern in CFB technique. F-D*ow*MAS, F-DMAS, and DAS were compared in terms of the resulting image quality metrics, Lateral resolution (LR), axial resolution (AR), contrast ratio (CR) and contrast-to-noise ratio (CNR), estimated from experiments on a commercially available tissue-mimicking phantom. The results demonstrate that F-D*ow*MAS improved the AR by 57.04% and 46.95%, LR by 58.21% and 53.40%, CR by 67.35% and 39.25%, and CNR by 44.04% and 30.57% compared to those obtained using DAS and F-DMAS, respectively. Thus, it can be concluded that the newly proposed F-D*ow*MAS outperforms DAS and F-DMAS. As an aside, we also show that the optimal weighting strategy can be extended to benefit DAS.

## Introduction

Ultrasound (US) imaging is widely used for clinical applications as it is a non-ionizing and real time modality. The beamformer is one of the most essential units of an US machine, which majorly determines the final reconstructed image quality. The primary purpose of the beamformer is to achieve a narrow main lobe with reduced side lobes, since the resolution is related to the width of the main lobe, and contrast is related to the level of side lobes^1^. Therefore, a beamformer is practically tuned to achieve a good tradeoff between these two^2^. Delay and sum beamformer is generally used in medical US imaging due to its simplistic implementation. However, it has its own limitations of lower image resolution outside the focal region and less off-axis interference rejection^2,3^. In literature, adaptive beamformers are proposed, which change the weights for the receive aperture dynamically based on the received data statistics to improve the image resolution, but with an increased computational complexity^4–8^. Also, methods based on using the spatial coherence have also been reported to improve the image quality^9–11^.

Few years ago, a non-linear beamforming technique called filtered delay multiply and sum (F-DMAS) beamforming was introduced for US imaging inspired from delay multiply and sum (DMAS) beamforming reported in microwave literature^12^. F-DMAS method was applied on plane wave imaging and synthetic transmit aperture (STA) techniques and it was demonstrated to improve the reconstructed image quality^13,14^. Later, to suppress the noise and artifacts further, an additional correlation process was introduced to F-DMAS and was named double-stage DMAS, thereby, improving the final reconstructed image quality^15^. Furthermore, a new method was proposed, which recursively applies the DMAS algorithm to improve the image quality^16^.

Further, an adaptive beamformer was proposed called p-DAS which resulted in improvement of image quality. It was also shown that, for p = 2, p-DAS is equivalent to DMAS^17^. Another research group proposed Baseband-DMAS (BB-DMAS), where the quality of the image is improved by utilizing pth root and pth power restoration operations on delayed and summed RF data, respectively^18^. The reason for improvement of image quality by F-DMAS over DAS was found to be the greater dependence of the image amplitude on signal coherence in the former compared to later^19,20^. Many works utilized this finding to improve the image quality, for e.g., sDMAS, 2D-DMAS, etc., which were developed by integrating coherence based beamformers with F-DMAS^20–24^.

Recently, weighted non-linear beamformers called filtered delay weight multiply and sum (F-D*w*MAS) and filtered delay Euclidian-weighted multiply and sum (F-D*ew*MAS) were proposed, where either an optimal weight or Euclidian-based weighting after the stage of multiplication was proposed for F-DMAS. It was shown that the quality of the reconstructed image improved for the data acquired using 2 receive synthetic aperture focusing technique (2R-SAFT) scheme using both F-D*w*MAS and F-D*ew*MAS and for data acquired using synthetic transmit aperture (STA) scheme using F-D*w*MAS^25–27^. Even though SA-based methods like STA or MSTA are proven to give better image quality along the depth, conventional focussed beamforming (CFB) is still widely used in the commercial scanners due to some practical advantages, such as, requiring less memory for data handling, lower beamforming complexity, thereby, reducing processing time and hardware requirement, better depth of penetration due to focussing in front of the transducer. Also, it gives best image quality at the focus, which can be flexibly set at desired region of interest and is one of the major advantages. Additionally, several US applications developed over years like pulse wave Doppler flow imaging, elastography, etc., were developed and proven successful for CFB scheme. Therefore, it appears advantageous to improve the image quality of CFB technique to derive greater impact.

In CFB using linear array transducer, a transmit sub-aperture with fixed focus is typically translated electronically from left to right through the array. Thus, in practice only a partial field of view benefits from having data from sub-aperture with symmetric transmit beam pattern. For e.g., in a 128 linear array transducer that operates with 64-active elements, only the region in the medium that falls between elements 32 and 96 will benefit from being insonified by the active sub-aperture with symmetric transmit beam pattern. Consequently, the practical transmit beam pattern changes for different transmissions since the beam must be steered asymmetrically, due to unavailability of sufficient number of elements when approaching the edges. This dictates the need to modify the weighted non-linear beamformers to make them optimal for CFB scheme.

In this paper, a new method named filtered delay optimally-weighted multiply and sum (F-D*ow*MAS) beamforming is proposed, which compensates for the above explained variations in the transmit beam, thereby, resulting in significantly improved final reconstructed image quality. Although the major interest was to improve the performance of the latest non-linear beamformer, the developed apodization optimization method is directly extendable to traditional DAS as well. This aspect is also reported here.

## Materials and methods

### Conventional focused beamforming (CFB) technique

**Figure 1:**
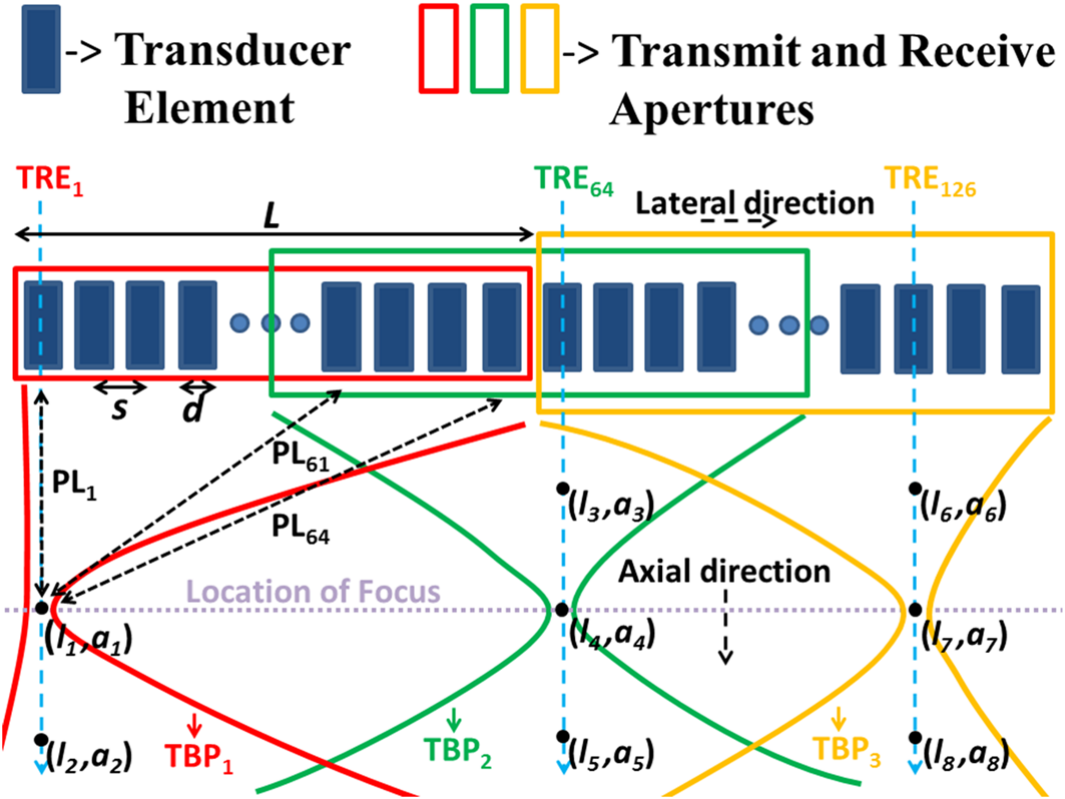
A schematic representing the changes in the transmit beam pattern (TBP) in the CFB scanning using a 128 element transducer, where, 65th transmit is symmetric around the aperture center compared to 1st and 126th transmits, which are skewed.

In Conventional focused Beamforming (CFB) technique, focused beams are used for imaging the medium. In CFB a set of transducer elements are excited (called as ‘transmit aperture’) to generate a beam that is focused at a particular depth. During reception, the set of elements receiving the returned echoes is referred to as a ‘receive aperture’. Later, the transmit aperture is scanned in lateral direction to acquire raw RF data, which is later beamformed, envelope detected, log compressed and finally displayed as B-mode image. There are different ways of scanning in CFB. In one such method, a transmit aperture whose size is much smaller compared to the size of the transducer is chosen such that only the scanning lines near the center, which has the same number of channels for all the transmit apertures, are used for imaging. However, this method either needs a larger transducer for scanning a given region or should compromise on the field of view. There are also variations where the number of transmit channels is reduced for the scan lines at the edges. However, the image quality will be degraded in the process due to less data available for beamforming. Further, the imaging depth will be compromised due to reduced total transmit energy. Furthermore, the changed size of the aperture for scan lines close to the edges, also introduces changes to the beampattern. There are also variations such as trapezoidal scanning, where the direction of the scanning lines is tilted (oblique scanning), which also results in image quality degradation due to the beampattern variations. In this paper we selected a CFB scan technique where the aperture chosen during both transmission and reception uses the maximum permitted active channel supported by the Ultrasound scanner hardware (i.e., 64 channel aperture on a 128-element transducer in our case). Since maximum possible aperture size is used in transmission, transmit energy and thereby the imaging depth is not compromised. Also, using maximum possible size during reception ensures that the SNR is not sacrificed due to missing data. The chosen CFB technique is illustrated in Fig. 1. The transmit aperture is scanned in lateral direction (indicated by red, green and oranges rectangles in Fig. 1, corresponding to different transmit receive events (TREs)) to acquire raw RF data, which is later beamformed, envelope detected, log compressed and finally displayed as B-mode image.

The transmit beam in CFB scheme is such that the focus is along the ‘line of sight’ of the transmit aperture center, when sufficient number of elements are available on either side, as shown in TRE_64_ of Fig. 1. If this is not the case, the beam is focused and steered as shown for TRE_1_ and TRE_126_ in Fig. 1. This is also the case with TRE_1_ to TRE_32_ and TRE_97_ to TRE_128_, where the transmit aperture remains static but the beam is steered, thus leading to huge variations in the transmit beam pattern for various transmissions, both in axial and lateral directions. This can be mathematically explained using Eq. (1), which gives the transmit beam pattern at any particular location $\left(l,a\right)$ when an Ultrasound beam is focused by an Array transducer made of rectangular elements^28^.

1 $$U\left(l,a\right) \alpha {L\, sinc}\left(\frac{ Ll }{\lambda a}\right) {s \,comb}\left(\frac{ sl }{\lambda a}\right){d\, sinc}\left(\frac{ dl }{\lambda a}\right),$$

where, $U$ is the intensity of beam, $\alpha$ represents “proportional to”, $l$ and $a$ are the spatial co-ordinates in lateral and axial directions, respectively, $L$ is the length of the array transducer, $\lambda$ is the wavelength, $s$ is the spacing between the elements and $d$ is the element width.

From both, Eq. (1) and Fig. 1, it can be observed that the intensity at a particular point (*l*_1_, *a*_1_) is dependent on the distance between the element and the point: Farther elements would contribute lesser compared to the nearer elements, due to attenuation. Therefore, at the point (*l*_1_, *a*_1_) the intensity contribution due to first element that is at a lesser path length (PL_1_) would be more, compared to sixty fourth element that is at a greater path length (PL_64_). In the same way, by analyzing path lengths between points (*l*_3_, *a*_3_), (*l*_4_, *a*_4_) and (*l*_5_, *a*_5_) and various transducer elements in transmit aperture, it can be observed that the intensity contribution of center element is highest compared to extreme elements, which is very different from the case of (*l*_1_, *a*_1_) and (*l*_2_, *a*_2_), where the contribution was highest from the first element, which is on the left extreme. Similarly, for points, (*l*_6_, *a*_6_), (*l*_7_, *a*_7_) and (*l*_8_, *a*_8_), the contribution would be highest from sixty second element, which is neither center nor an extreme element. Also, due to attenuation the contribution from various elements would change with respect to depth. For example, even though the contribution from the first element to points (*l*_1_, *a*_1_) and (*l*_2_, *a*_2_) is highest compared to other elements, it would be higher for (*l*_1_, *a*_1_) compared to (*l*_2_, *a*_2_), due to depth dependent attenuation. In summary, the contributions from different elements to different points in the medium for different transmissions would vary drastically based on factors like location of point in the medium and the distance of the point from various elements. Further, the wavelength ($\lambda$) would change with distance, due to frequency shifts caused by attenuation. From all the above observations, it can be concluded that the transmit beam pattern varies at different points in the imaging plane. Due to these variations, even though the focus is located at the same depth, ‘width of the beam at focus’ and ‘depth of focus’ changes for different transmits, which affects the resolution.

### Linear (DAS), non-linear (F-DMAS) beamforming methods

In DAS beamforming, to reconstruct a point in the medium, the raw RF data acquired over the receive aperture is delayed making them in phase and then summed. Whereas, in DMAS it is combinatorially coupled and multiplied before being summed^12^. Equations (2) and (3) gives the final beamforming equations of DAS and DMAS.

2 $$\text{DAS}\left(\text{t}\right)=\sum_{i=1}^{n}{w}_{i}\left(t\right){d}_{i}\left(t\right),$$

3 $$\text{DMAS}(\text{t})=\sum_{i=1}^{n-1}\sum_{j=i+1}^{n}{d}_{i}\left(t\right){d}_{j}\left(t\right).$$

where, *d*_*i*_*(t)* and *d*_*j*_*(t)* are the delayed RF signals received by the *i*th and *j*th transducer elements, *w*_*i*_*(t)* is the apodization window coefficient multiplied to *d*_*i*_*(t),* and *n* is the number of transducer elements in receive aperture.

Further, additional steps of sign preservation, dimensionality reduction and bandpass filtering were introduced in DMAS to develop filtered DMAS (F-DMAS)^12^.

### Weighted non-linear (F-DwMAS and F-DewMAS) beamforming methods

In the recently proposed filtered delay weight multiply and sum (F-D*w*MAS) and filtered delay euclidian-weighted multiply and sum (F-D*ew*MAS) beamforming methods, an extra stage of weighting is introduced after the delay stage. In F-D*w*MAS, a set of window coefficients raised to the power of ‘r’ are multiplied to the aperture data, where ‘r’ can be used as a tuning parameter to control image quality. Whereas, in F-D*ew*MAS, the combinatorial combinations of the window coefficients obtained after the multiplication stage are raised to the power of ‘Euclidian distance’^26^. In general, these operations can be mathematically represented as in Eq. (4). The sign of the signal is preserved in Eq. (5), while Eq. (6) gives the final beamforming equation for either F-D*w*MAS or F-D*ew*MAS depending on the ‘*power*’ chosen.

4 $$x\left(i,j\right)=d\left(i\right)d\left(j\right){\left(w\left(i\right)w\left(j\right)\right)}^{power},$$

5 $$y\left(i,j\right)=sign\left(x\left(i,j\right)\right).\sqrt{|x\left(i,j\right)|},$$

6 $$DewMAS\left(.\right) \,or\, DwMAS(.)=\sum_{i=1}^{\frac{{n}^{2}-n}{2}}y\left(.\right),$$

where, $power=r \left(\text{for D}w\text{MAS}\right)\text{ or }\left|i-j\right| (\text{for D}ew\text{MAS})$ when *d(i)* and *d(j)* are the delayed RF signals received by the ith and jth transducer elements, respectively. x(i,j) is the weighted combinatorially coupled data, y(i,j) preserves the sign and finds the square root x(i,j) and n is the number of transducer elements in receive aperture.

Further detailed explanation of F-D*w*MAS and F-D*ew*MAS beamforming methods can be found in reference Vayyeti and Thittai^26^.

### Filtered delay optimally-weighted multiply and sum (F-DowMAS) beamforming

It was explained in the previous section that the transmit beam pattern varies in CFB scheme. The proposed filtered delay optimally-weighted multiply and sum (F-D*ow*MAS) beamformer incorporates compensation for the changes in the transmit beam pattern caused due to the position $(l,a)$ and wavelength $(\lambda )$ (in Eq. (1)) by optimizing the apodization function at every imaging location $(l,a$). The developed F-D*ow*MAS beamformer tries to reduce the width of the main lobe of receive beam pattern while simultaneously reducing the side lobe levels. Since image resolution is related to the width of the main lobe and image contrast is related to the level of side lobes, F-D*ow*MAS can improve the overall image quality. F-D*ow*MAS beamformer tries to achieve this by minimizing Eq. (7), where, axial resolution (AR), lateral resolution (LR), contrast ratio (CR) and contrast to noise ratio (CNR) are used as optimizing parameters. The numerator of Eq. (7) consists of resolution terms, which takes care of reducing the main lobe in both axial and lateral direction, whereas, the denominator consisting of contrast terms take care of reducing the level of side lobes. F-D*ow*MAS can be mathematically described using Eqs. (7)–(11), where, Eqs. (7)–(9) compensates for the variations in the beam pattern.

7 $$\text{m}\left(\text{p},\text{r}\right)=\text{min}\left(\frac{\text{ AR}\left(\text{p},\text{r}\right).\text{LR}\left(\text{p},\text{r}\right) }{\left|\text{CR}\left(\text{p},\text{r}\right)\right|.\text{CNR}\left(\text{p},\text{r}\right)}\right),$$

8 $$\text{R}\left(\text{p}\right)=\text{r}\ni {^{\prime}}{\text{m}}{^{\prime}}\text{ is minimum w}.\text{r}.{\text{t}} {^{\prime}}{\text{r}}{^{\prime}} @{ {^{\prime}}}{\text{p}}{^{\prime}},$$

9 $$x\left(i,j\right)=d\left(i\right)d\left(j\right){\left(w\left(i\right)w\left(j\right)\right)}^{R(p)},$$

10 $$y\left(i,j\right)=sign\left(x\left(i,j\right)\right).\sqrt{|x\left(i,j\right)|},$$

11 $$DawMAS\left(p\right)=\sum_{i=1}^{\frac{{n}^{2}-n}{2}}y\left(.\right),$$

where, *AR* and *LR* are computed as full width at half maximum (at − 6 dB level) across the Axial and Lateral profiles of point scatterrers, *CR* and *CNR* are computed as explained in reference 26, p represents the imaging point being operated, *r* is the power variable varied over a wide range (e.g., 0 to 200, in discrete steps and later interpolated to attain resolution of 0.001), *m* is the function to be minimised, $\text{R}\left(\text{p}\right)$ is the adaptive power matrix computed on the whole raw RF data acquired at full sampling frequency, *d(i)* and *d(j)* are the delayed RF signals received by the *i*th and *j*th transducer elements, respectively. *x(i,j)* is the weighted combinatorially coupled data, *y(i,j)* preserves the sign and finds the square root *x(i,j),* and *n* is the number of transducer elements in receive aperture.

It can be observed from the above equations that R(p) must be estimated, which requires calculation of image quality metrics from the image. This estimation of R(p) operates similarly to a closed loop feedback system, to optimize the weights and make them more adaptive to the variations in the beam shape and intensity. However, this process being computationally intensive may not be suitable for real-time operation. Instead, we propose computing these adaptive weights (Eq. (8)), as a calibration step before hand. This is practical since it can be done once for the chosen transducer and system configuration either through experiments on a calibrated test phantom or in simulation. The estimated R(p) can then be stored and used in Eq. (9). Thereafter, Eqs. (10) and (11) are used to perform D*ow*MAS reconstruction.

### Estimation of the adaptive power matrix, R, in simulation

**Figure 2:**
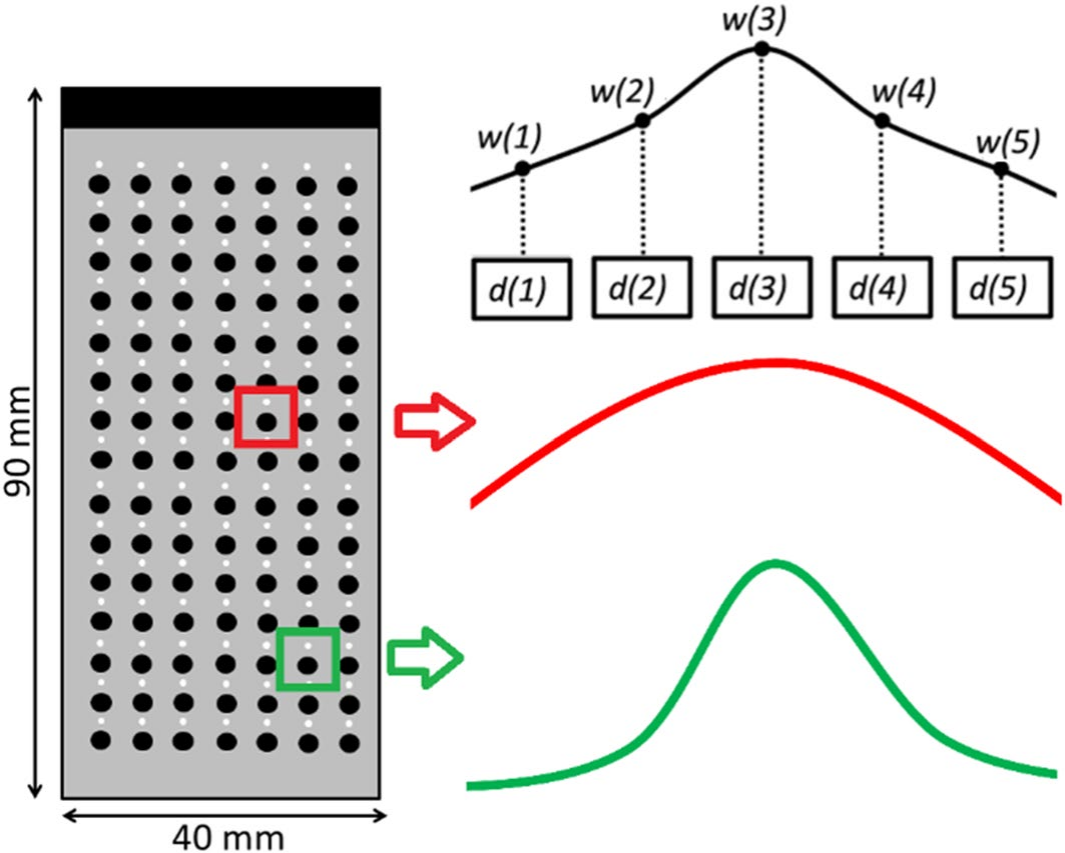
The cross-section view of the simulated phantom consisting of strong point reflectors (white circles), no scattering regions (black), and scattering region (grey). The phantom geometry, along with dimensions, is also shown. An illustration of the apodization function at two different points in the imaging medium shaped by R(p) to compensate for beampattern variations is also provided.

For the purposes of this study, we estimated R(p) from simulation and evaluated the performance of F-D*ow*MAS beamformer in experiments. Here, a computer test phantom was generated using Field II software^29,30^. The phantom contained both point scatterers and cysts (2 mm diameter), which were equally spaced and arranged in 15 rows and 7 columns to cover the entire imaging region as shown in Fig. 2. The arrangement and spacing of the point scatterers and cysts were selected such that the side lobes of one scatterer will not interfere with the main lobe of the neighbouring scatterers, since this would corrupt the AR and LR computations. Also, the size of the cysts was selected in such a way to incorporate as many cysts as possible while having enough background region for CR and CNR computations for any of the cyst. This computer phantom was imaged with a simulated array transducer using the CFB scheme with the parameters as listed in Table 1. The simulated array transducer was as close as possible to the L11-5v (Verasonics Inc., WA.USA) transducer used in the experiments. Gaussian white noise was added to the simulated data so that the SNR of the acquired RF data after the addition of noise was at 40 dB. This RF data was subject to the process described earlier (Eqs. (7)–(11)) to find the optimal adaptive weights ‘R’, which are assigned to the midpoint of the line joining the centers of point scatterer and the cyst. At other imaging locations interpolated values were generated.

**Table 1:** Transducer parameters used in simulation.

Parameter	CFB scheme
Number of elements (N_T_)	128
Active elements (*n*)	64 (TX), 64 (RX)
Inter-element spacing	0.3 mm
Element width	0.275 mm
Element height	4 mm
Center frequency	8 MHz
Sampling frequency	31.25 MHz
Focus	40 mm

### Example numerical analysis to explain the role of adaptive power matrix, R, in compensating for beam pattern variation

Consider two points p_1_ (close to focus) and p_2_ (away from focus) that are within the locations highlighted by red and green boxes in Fig. 2, which were insonified by a focused beam transmitted through a transmit aperture consisting of 5 transducer elements. Assuming that 5 raw RF data points (d_1_ to d_5_, acquired by receive aperture consisting of same 5 transducer elements) are used to reconstruct these points by F-D*ow*MAS beamformer, a total of 10 combinations will be generated as shown in Eq. (12). Where, w_1_ to w_5_ are the window coefficients multiplied to d_1_ to d_5_ as shown in Fig. 2, and R(p_1_) is the value of power at location p_1._

12 $${C}_{1}\left(i,j\right)=\left[\begin{array}{llll}{d}_{1}{d}_{2}{{(w}_{1}{w}_{2})}^{R\left({p}_{1}\right)}& {d}_{1}{d}_{3}{{(w}_{1}{w}_{3})}^{R\left({p}_{1}\right)}& {d}_{1}{d}_{4}{{(w}_{1}{w}_{4})}^{R\left({p}_{1}\right)}& {d}_{1}{d}_{5}{{(w}_{1}{w}_{5})}^{R\left({p}_{1}\right)}\\ {d}_{2}{d}_{3}{{(w}_{2}{w}_{3})}^{R\left({p}_{1}\right)}& {d}_{2}{d}_{4}{{(w}_{2}{w}_{4})}^{R\left({p}_{1}\right)}& {d}_{2}{d}_{5}{{(w}_{2}{w}_{5})}^{R\left({p}_{1}\right)}& -\\ {d}_{3}{d}_{4}{{(w}_{3}{w}_{4})}^{R\left({p}_{1}\right)}& {d}_{3}{d}_{5}{{(w}_{3}{w}_{5})}^{R\left({p}_{1}\right)}& -& -\\ {d}_{4}{d}_{5}{{(w}_{4}{w}_{5})}^{R\left({p}_{1}\right)}& -& -& -\end{array}\right].$$

**Figure 3:**
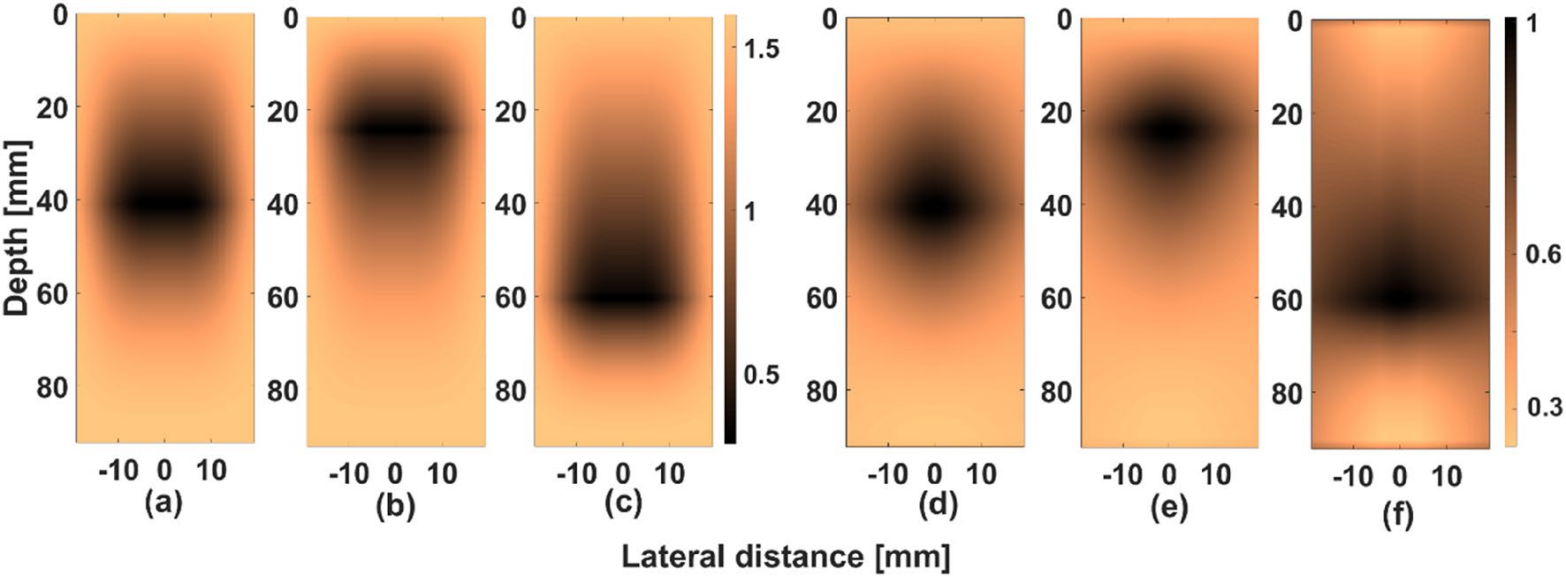
Image of ‘R’ matrix obtained for different transmit focal depth settings of (**a**) 40 mm, (**b**) 25 mm and (**c**) 60 mm in CFB technique that is used in F-D*ow*MAS. The corresponding normalized amplitude map computed by taking maximum intensities in their transmit beams (along lateral direction) are shown in (**d–f**), respectively.

Now, assuming the following values for window coefficients (Hanning window) and powers (taken from Fig. 3) we have $${w}_{1}=0.1, { w}_{2}=0.5, {w}_{3}=1, {w}_{4}=0.5,{ w}_{5}=0.1, R\left({p}_{1}\right)=0.5 (close to focus) , R\left({p}_{2}\right)=1$$ (away from focus). Substituting these values in Eq. (12), we get values as shown in Eq. (13) for point p_1_

13 $${C}_{1}\left(i,j\right)=\left[\begin{array}{llll}{d}_{1}{d}_{2}\left(0.22\right)& {d}_{1}{d}_{3} \left(0.31\right)& {d}_{1}{d}_{4} \left(0.22\right)& {d}_{1}{d}_{5}\left(0.1\right)\\ {d}_{2}{d}_{3}\left(0.707\right)& {d}_{2}{d}_{4}\left(0.5\right)& {d}_{2}{d}_{5}\left(0.22\right)& -\\ {d}_{3}{d}_{4}\left(0.707\right)& {d}_{3}{d}_{5}\left(0.31\right)& -& -\\ {d}_{4}{d}_{5}\left(0.22\right)& -& -& -\end{array}\right] .$$

Similarly, for point p_2_, the computed values are shown in Eq. (14)

14 $${C}_{2}\left(i,j\right)=\left[\begin{array}{llll}{d}_{1}{d}_{2}\left(0.05\right)& {d}_{1}{d}_{3} \left(0.1\right)& {d}_{1}{d}_{4} \left(0.05\right)& {d}_{1}{d}_{5}\left(0.01\right)\\ {d}_{2}{d}_{3}\left(0.5\right)& {d}_{2}{d}_{4}\left(0.25\right)& {d}_{2}{d}_{5}\left(0.05\right)& -\\ {d}_{3}{d}_{4}\left(0.5\right)& {d}_{3}{d}_{5}\left(0.1\right)& -& -\\ {d}_{4}{d}_{5}\left(0.05\right)& -& -& -\end{array}\right] .$$

From Eqs. (13) and (14), it can be noted that, ${C}_{1}\left(\text{2,1}\right)={1.4 C}_{2}\left(\text{2,1}\right) ; {C}_{1}\left(\text{1,4}\right)={10 C}_{2}\left(\text{1,4}\right)$.

It can be observed that the Beamformer gave more weightage to all the combinations involved in reconstruction of point p_1_ compared to those involved in reconstruction of p_2,_ since the SNR of the signal received from focal region is greater due to relatively stronger insonification compared to region away from the focus. When the point is away from the focus, the beamformer assigns disproportionately lower weightage to the points far from the “line of sight” of CFB. For example, ${C}_{2}\left(\text{1,4}\right)$ was given 10 times lower weightage compared to ${C}_{1}\left(\text{1,4}\right)$, since either ${d}_{1}$ or ${d}_{5}$ or both will be the farthest from “line of sight” depending on the transmission (i.e., if it is symmetric, ${d}_{1}$ and ${d}_{5}$ are both farthest, if it is right skewed, ${d}_{5}$ is the farthest and if it left skewed, ${d}_{1}$ is the farthest) and would lead to a larger attenuation (exponential decay in signal) in both transmission and reception and therefore lower SNR. Whereas, for ${C}_{2}\left(\text{2,1}\right)$ the weightage was reduced only by a factor of 1.4, since, ${d}_{2}$ and ${d}_{3}$ are relatively closer to “line of sight” irrespective of transmission, so the attenuation levels are lower compared to the ${C}_{1}\left(\text{1,4}\right)$ and ${C}_{2}\left(\text{1,4}\right)$ case. Also, from Fig. 3, the weightage is related to intensity of transmit beam at a particular location, which takes care of the intensity variations due to skewness (or left–right symmetry) of the beam by changing weightage accordingly. In this manner, the beamformer changes the weightage based on the location of the points in the medium, thus compensating for beampattern variations. The other way to look at this process is that, the shape of the apodization function is changed at every location (as shown in Fig. 2) giving more weightage to signals with better SNR during beamforming thus resulting in better image quality.

In this paper, Hanning was chosen as the window function without loss of generality; however, one can select any other window functions as well. The filters applied for various methods are listed in Table 2, along with the cut-off frequencies, where the transmit frequency was 8 MHz. The post filters were applied to the interpolated data since the sampling frequency was 31.25 MHz.

**Table 2:** Frequency cutoffs of the BP-filter windows for various methods.

Method	Pre-filter on RF signals (MHz)	Post-filter after beamforming (MHz)
DAS/D*ow*AS	5–11	5–11
F-DMAS/F-D*w*MAS/F-D*ew*MAS/F-D*ow*MAS	5–11	8–20

### Delay optimally-weighted and sum (DowAS) beamformer

It is reasonable at this time to expect that the developed apodization optimization method can be readily adapted to also DAS beamformer. Therefore, we incorporated this strategy to develop delay optimally-weighted and sum beamformer (D*ow*AS) and report it in comparison with the other methods in simulations and experiments.

### Data involved in beamforming process of all the beamformers and complexity reduction for CFB

Consider a CFB case, where the number of receive elements is equal to $n$*.* Now, to reconstruct one pixel in any reconstructed RF A-line DAS makes use of a maximum of $n$ raw RF data points. Whereas, F-DMAS/F-D*w*MAS/F-D*ew*MAS/F-D*ow*MAS handles $\left(0.5\times n\times \left(n-1\right)\right)$ raw RF data points in the process. In a similar fashion, for an N_T_-element transducer, a total of N_T_ reconstructed RF A-lines are formed, using the RAW RF data acquired from different transmit-receive events and are stacked next to each other to form the final reconstructed image. Since the data involved in beamforming is high, to reduce the processing and computational complexity and resources, F-D*ow*MAS beamformer was implemented utilizing matrix operation based real time method proposed by Jeon et al.^31^.

### Phantom experiments

Experimental data were acquired using Vantage 64 (Verasonics Inc., WA.USA) scanner with L11-5v (Verasonics Inc., WA, USA) transducer operating at 8 MHz sampled at 31.25 MHz. Experimental imaging studies were performed on commercially available ‘Multi-Purpose, Multi Tissue Ultrasound phantom (Model 040GSE)’ (CIRS, 2428 Almenda Suite, Norfolk, Virginia 23515, USA).

All the settings for the experiments, including the number of active elements during transmit and receive, location of focus, etc., were the same as that of simulations (see Table 1).

### In-vivo data

The developed methods were tested on an example in vivo data from the “Challenge on Ultrasound Beamforming with Deep Learning (CUBDL)” data set available online^32–34^. The data was acquired by taking informed consent form from the volunteers after the approval of the Johns Hopkins Medicine Institutional Review Board (IRB00127110). All experiments were performed in accordance with relevant guidelines and regulations. Specific permission was obtained to use the data for this publication.

## Results and discussion

Figure 3 shows the map of the window coefficients (‘*r*’) that is used in F-D*ow*MAS over the entire imaging region for three different settings of the focal depth in a CFB technique. It can be observed that the value of ‘r’ varies both in the axial and lateral directions, due to the variations in the transmit beam pattern. Also, the variations in the pattern are minimal with respect to the change in the focal depth, except for a spatial shift, which can be used to make the F-D*ow*MAS beamformer adaptive to change in the focal location. Figure 3d–f shows the corresponding normalized amplitude maps of the transmit beam. It can be observed from Fig. 3 that the adaptive power matrix ‘R’ has a close relation to amplitude levels of the transmit beam. It is clear from Fig. 3 that weightage given suitably compensates for the variations in the beampattern.

**Figure 4:**
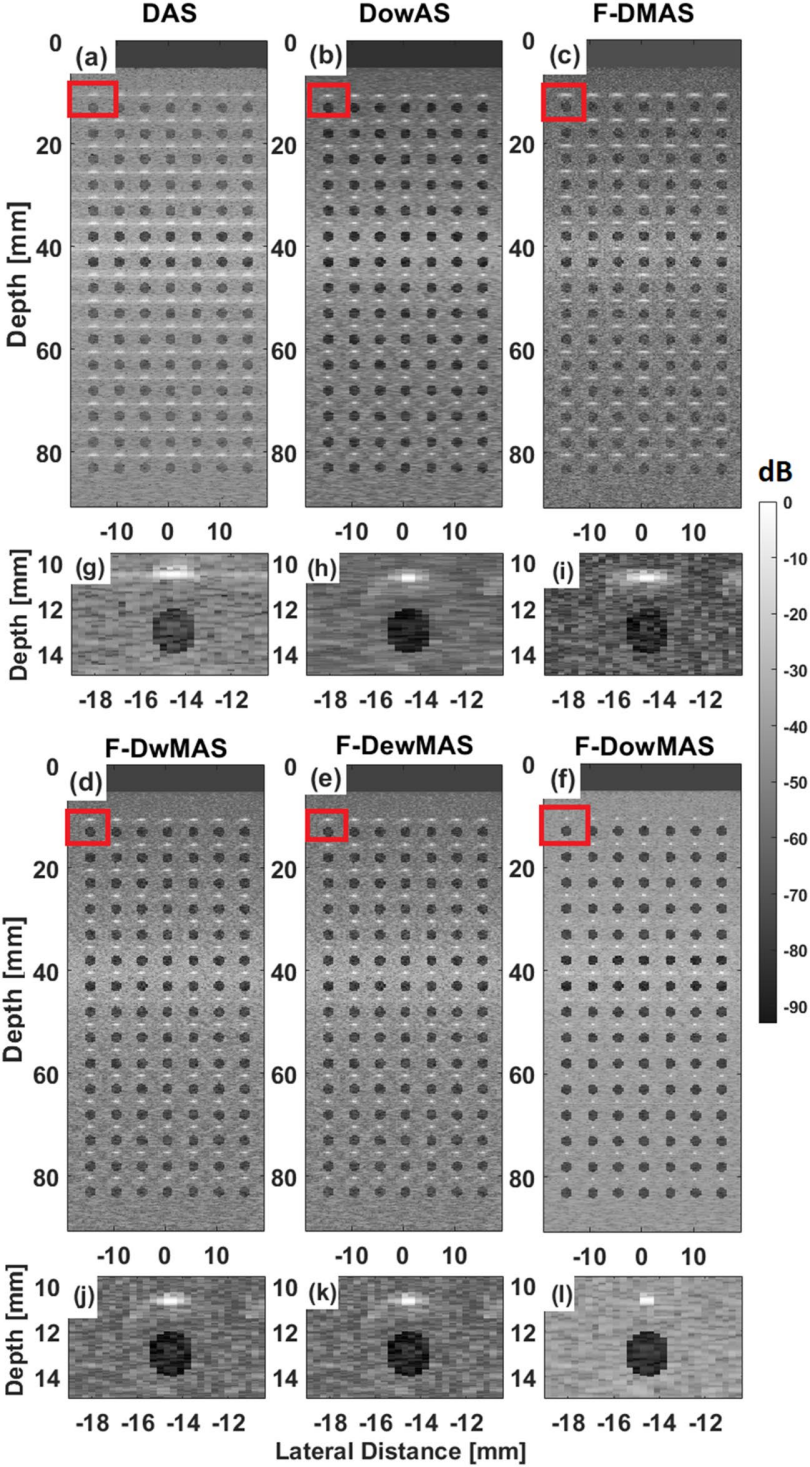
B-mode images of the computer phantom reconstructed using the different beamformers (**a–f**), wherein, the data were acquired using CFB technique. A zoomed region (indicated by red rectangle) from within these B-mode images are also shown in (**g–l**), respectively.

**Figure 5:**
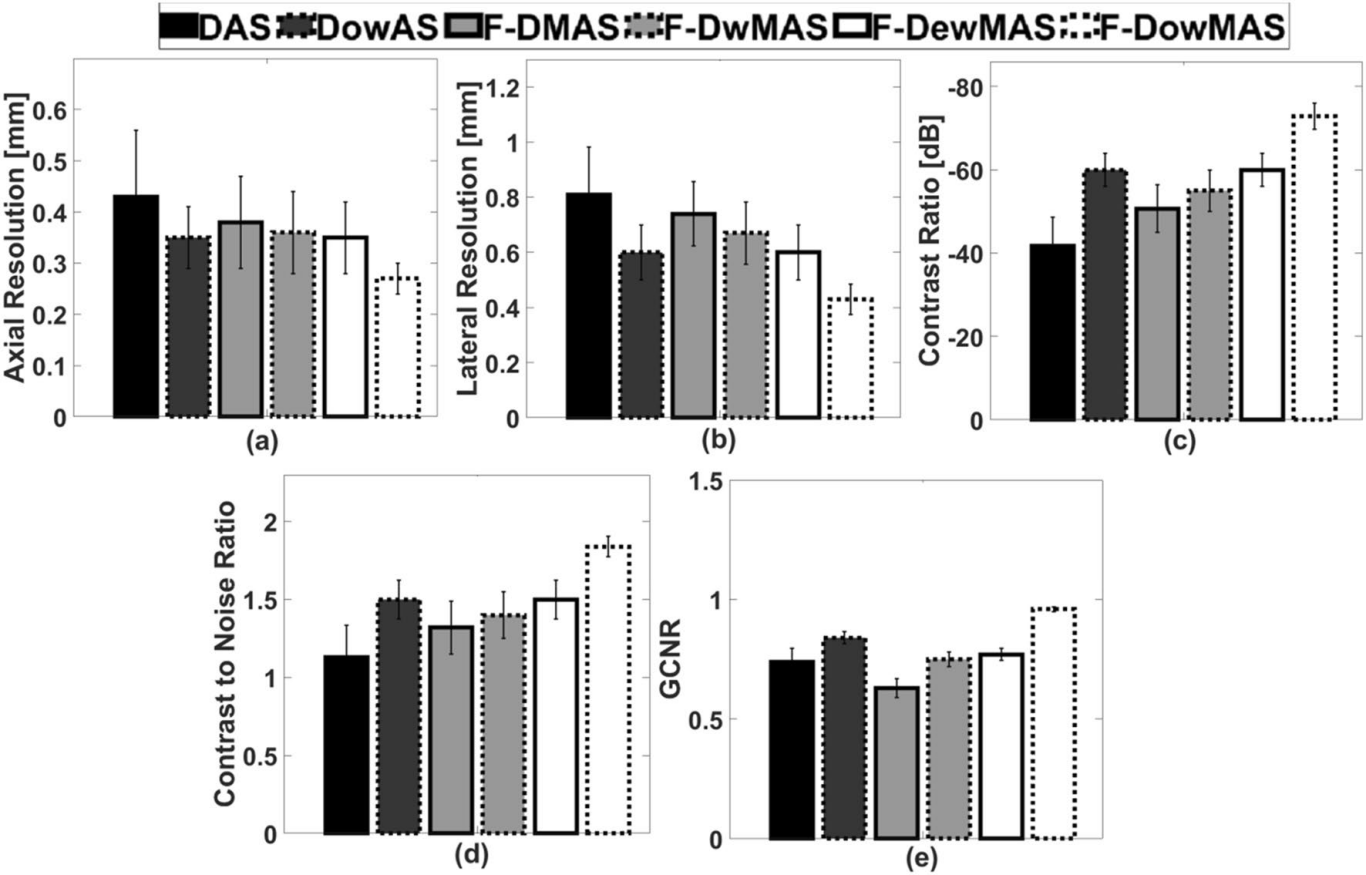
Bar plots showing a comparison of the different image quality metrics (mean and standard deviation over all 105 cysts and 105 point scatters) obtained in simulations.

Figure 4 shows the B-mode images of the computer-generated phantom with points and cysts reconstructed using various reconstruction methods. Visually, the image reconstructed using F-D*ow*MAS appears to be the best having the least spread of the points. Further, from the zoomed cyst region shown in images of Fig. 4, it can be observed that not only does F-D*ow*MAS yields the tightest point spread, but also has the least noise level inside the cyst compared to those that are reconstructed using other methods. The developed D*ow*AS has lower point spread and lower noise level inside cyst compared to DAS and F-DMAS respectively. Furthermore, the quantitative image quality metrics of AR, LR, CR and CNR estimated from the images obtained using F-D*ow*MAS are superior compared to those obtained using other beamformers as observed in Fig. 5. Since GCNR is more robust metric compared to CNR, GCNR values are also computed and reported in Fig. 5^35,36^. The developed F-DowMAS beamformer has a better GCNR value compared to other methods.

**Figure 6:**
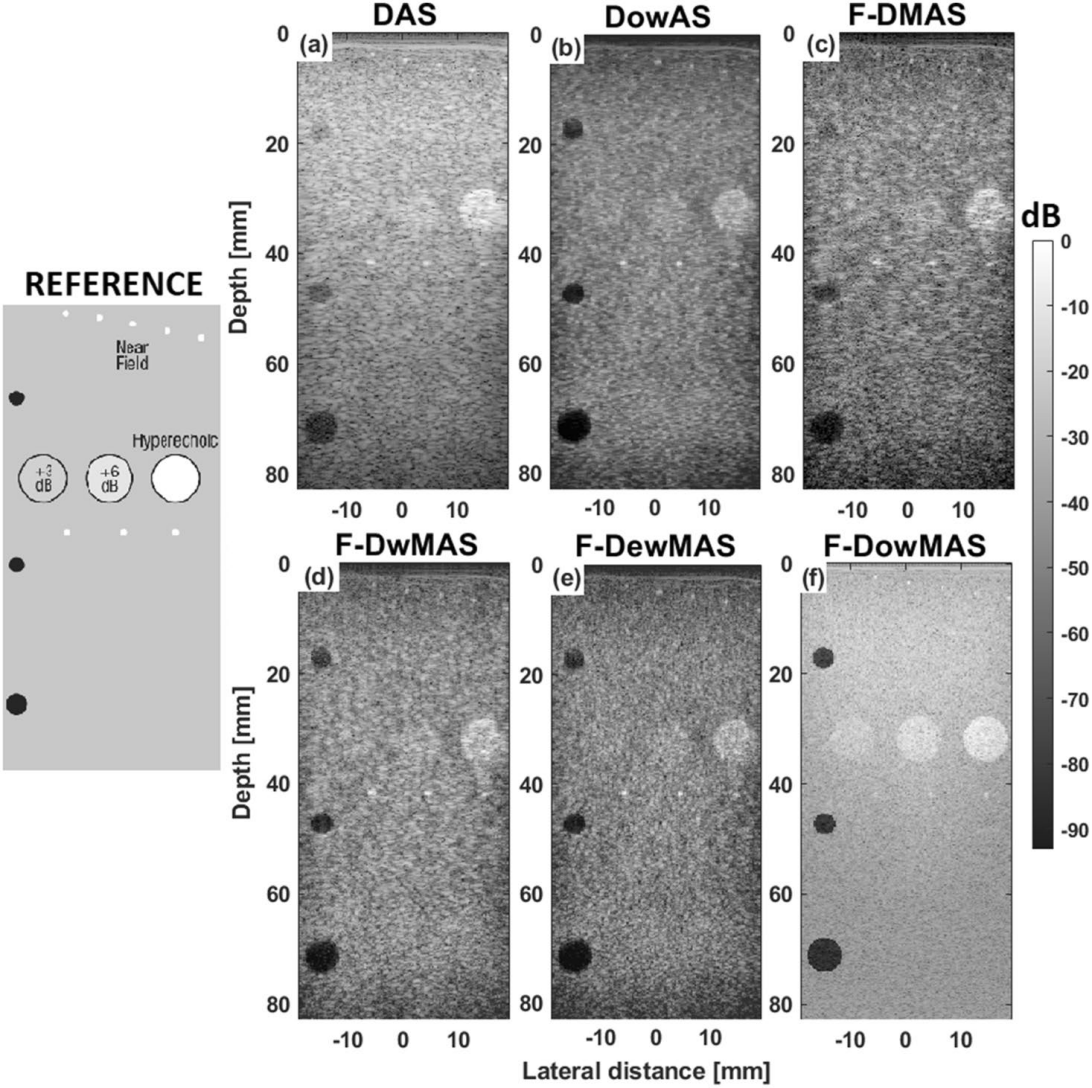
B-mode images of the CIRS phantom reconstructed using the different beamforming methods for data acquired using CFB.

Figure 6 shows the B-mode images from experiments performed on CIRS phantom. The images obtained using the different reconstruction methods are compared. It can be observed that the image reconstructed using F-D*ow*MAS not only has the least point spread, but also is visibly more comparable to the reference image in Fig. 6 than those images reconstructed using other methods. Specifically, the reconstruction of the high scattering regions, + 3 dB and + 6 dB, is much better visualized in image reconstructed by F-D*ow*MAS compared to all other methods, due to the adaptive weighting strategy that compensated for transmit beam variations.

**Figure 7:**
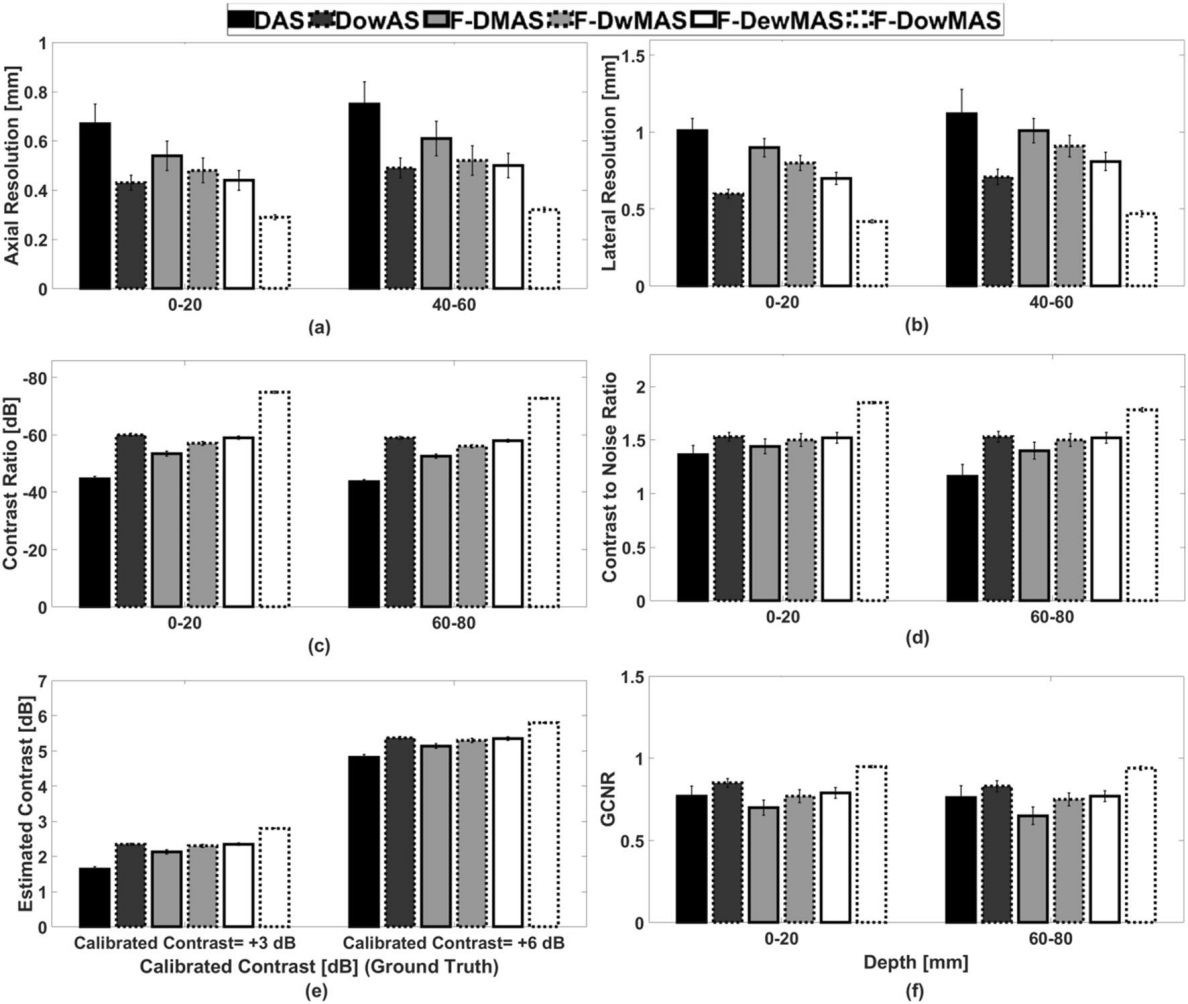
Bar plots showing a comparison of the different image quality metrics for various methods in experiments.

Figure 7 shows the mean and standard deviations of the estimated AR, LR, CR, CNR and GCNR values obtained from 12 independent experimental renditions.

For ready comparison, the calibrated reference contrast value is also listed alongside the estimated contrasts. All the metrics were computed from the point scatterers, cysts and calibrated targets located in both near and far fields. From Fig. 7, it can be observed that AR, LR, CR, CNR and GCNR estimated from images reconstructed using F-D*ow*MAS are better compared to those from images reconstructed using other methods. The developed D*ow*AS has lower point spread and lower noise level inside cyst compared to DAS and F-DMAS respectively. Further, from Fig. 7e it can be observed that the estimated contrasts are the closest to the calibrated ground truth contrasts (i.e., + 3 dB and + 6 dB) in the case of F-D*ow*MAS. The reason for the improvement is because of added imaging point based adaptive weighting, which reduces the contributions of the point spread, which influences the signal to noise ratio (SNR) levels.

**Figure 8:**
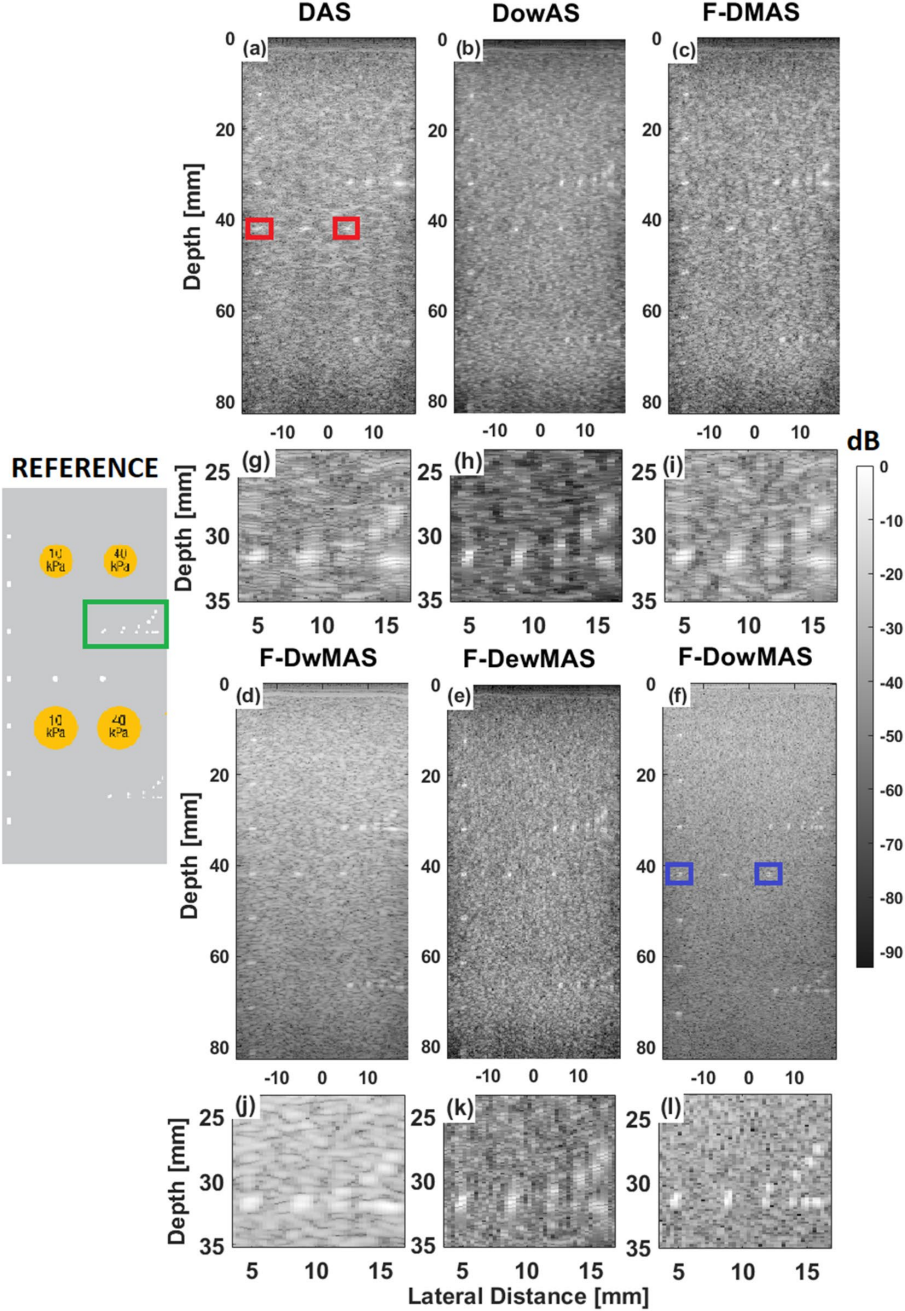
B-mode images of the CIRS phantom imaged at a different location containing point scatterers for resolution assessment reconstructed using the different beamforming methods. A zoomed around the resolution point scatterers (indicated by green rectangle in “REFERENCE”) from the respective B-mode images are also shown.

Figure 8 shows the B-mode images obtained from imaging experiments on CIRS phantom imaged at a different location. It can be observed from Fig. 8g–f that the point scatterrers are clearly separable in the images reconstructed with F-D*ow*MAS, unlike those that are reconstructed using other methods. This demonstrates an improvement in both AR and LR. In addition, it can be observed that the most point scatterers are well distinguishable from background only in the case of F-D*ow*MAS, which demonstrates an improvement in the contrast (CR and CNR). Results from experiments on phantom show that the proposed F-D*ow*MAS resulted in improvements of AR by 57.04% and 46.95%, LR by 58.21% and 53.40%, CR by 67.35% and 39.25% and CNR by 44.04% and 30.57% compared to those obtained using DAS and F-DMAS, respectively.

**Figure 9:**
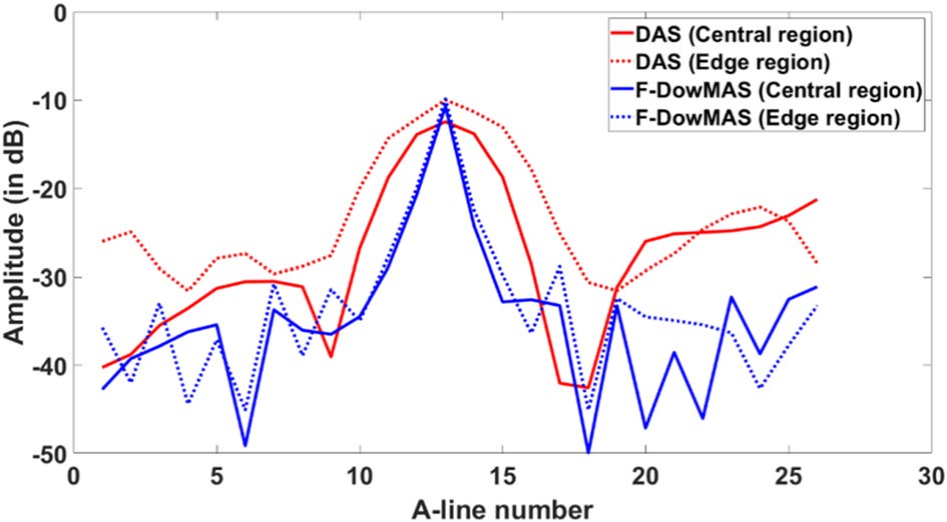
Lateral profiles taken across two point scatterrers (enclosed by red and blue rectangles in Fig. 8a,f) one from central and the other from edge in the images reconstructed by DAS and F-D*ow*MAS beamformers.

In order to better illustrate the benefit of the proposed method the lateral profiles of two points (enclosed by red and blue rectangles in Fig. 8a,f, respectively) are compared in Fig. 9. It can be observed that, in DAS reconstructed image with standard Hanning apodization, the point spread is more in the edge compared to the central region. Whereas, in the developed F-D*ow*MAS beamformer both the points have almost the same width due to beampattern compensation. Also, the improvement is more for the point present in the edge compared to that located in the central region, which demonstrates that the developed F-D*ow*MAS beamformer was able to compensate for variations in the left–right symmetry.

As explained earlier, DAS makes use of a maximum of $n$ RF data points, whereas F-DMAS/F-D*w*MAS/F-D*ew*MAS/F-D*ow*MAS handles a maximum of $\left(0.5\times n\times \left(n-1\right)\right)$ RF data points in the process to reconstruct one pixel in the final beamformed image, where *n* is the number of active receive elements. In this work, a transducer array consisting of 128 elements was used, and the active receive aperture for CFB was 64. Therefore, to reconstruct one pixel DAS makes use of 64 RF data points, whereas, F-DMAS, uses 2016 RF data points, where the additional data points are generated from the existing data. As stated earlier, the amount of data involved in the DMAS reconstruction is huge, and one may expect an improvement in the image quality. However, the results demonstrate that by exploiting appropriate weighting for each data one can achieve even better image quality.

Overall, F-D*ow*MAS have shown to improve the reconstructed image quality compared to DAS and F-DMAS. Also, since F-D*ow*MAS is adaptive to change in the focal depth, which is the major variable for clinical imaging, it makes it possible to immediately transfer this beamformer onto existing US machines by calibrating it for other parameters like transducer type, element pitch, bandwidth, active aperture size and excitation voltage etc., which are fixed in clinical applications. Perhaps, recently proposed generalized CNR (GCNR) can be used to optimize F-D*ow*MAS, which is left open for a future study^35^.

**Figure 10:**
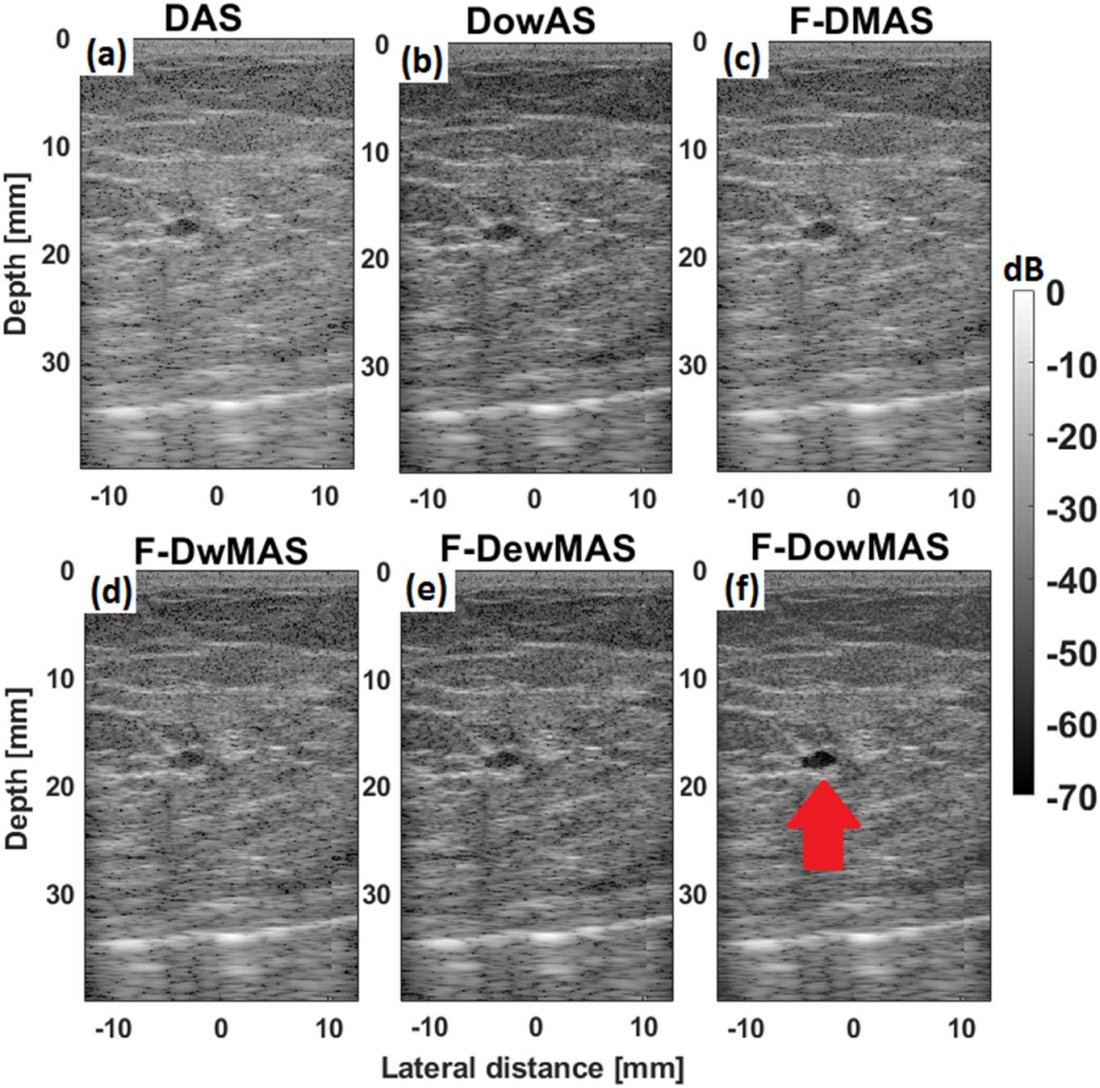
B-mode images of the example in vivo data reconstructed using the different beamformers. The red color arrow points to the cyst.

An in vivo example from CUBDL dataset, acquired from breast of a patient volunteer using L8–17 Transducer operating at a center frequency of 12.5 MHz using CFB and beamformed using the different methods is shown in Fig. 10 (Note: The R matrix for F-D*ow*MAS and D*ow*AS was recomputed in simulation to correspond to the acquisition parameters of the data). It can be observed that the proposed F-D*ow*MAS yields a visibly superior image quality compared to the rest. Specifically, the cyst is visible with better contrast compared to the background in the image reconstructed using F-D*ow*MAS.

## Conclusion

A novel optimally-weighted non-linear beamformer called F-D*ow*MAS was introduced. The results demonstrate clearly that the F-D*ow*MAS method significantly improved the image quality in terms of Axial Resolution, Lateral Resolution, Contrast, and Contrast to Noise Ratio compared to that obtained by either DAS or F-DMAS for CFB technique.
